# The formation of source memory under distraction

**DOI:** 10.1186/1744-9081-10-40

**Published:** 2014-10-24

**Authors:** Heekyeong Park, Fernando Leal, Cheryl Abellanoza, James D Schaeffer

**Affiliations:** Department of Psychology, University of Texas at Arlington, Arlington, TX 75069-19528 USA

**Keywords:** Source memory, Distraction, fMRI, Hippocampus, Encoding

## Abstract

**Background:**

It is vital to select and process relevant information while restraining irrelevant information for successful retrieval. When multiple streams of information are concurrently present, the ability to overcome distraction is very crucial for processing relevant information. Despite its significance, the neural mechanism of successful memory formation under distraction remains unclear, especially with memory for associations. The present fMRI study investigated the effect of distraction due to irrelevant stimuli in source memory.

**Methods:**

In the MR scanner, participants studied an item and perceptual context with no distractor, a letter-distractor, or a word-distractor. Following the study phase, a source recognition test was administered in which participants were instructed to judge the study status of the test items and context of studied items. Participants’ encoding activity was back-sorted by later source recognition to find the influence of distractors in subsequent memory effects.

**Results:**

Source memory with distractors recruited greater encoding activity in the left dorsolateral prefrontal cortex, and the bilateral inferior temporal gyrus/fusiform cortex, along with the left posterior hippocampus. However, enhanced activity in the left anterior ventrolateral prefrontal cortex and the left parahippocampal cortex predicted successful source memory regardless of the presence of a distractor.

**Conclusions:**

These findings of subsequent memory effects suggest that strong binding of the item-context associations, as well as resistance to interference, may have greater premium in the formation of successful source memory of pictures under distraction. Further, attentional selection to the relevant target seems to play a major role in contextual binding under distraction by enhancing the viability of memory representations from interference effects of distractors.

**Electronic supplementary material:**

The online version of this article (doi:10.1186/1744-9081-10-40) contains supplementary material, which is available to authorized users.

## Background

As information processing under distraction has become ubiquitous in modern society, processing relevant information from multiple streams of incoming information constitutes an important aspect of human cognition. Source memory involves the binding of contextual details for a specific event so that a new event can be distinguished from other similar events. Source memory is also considered to be supported by recollection, which is a more effortful process [1, 2]. Therefore, successful formation of source memory while filtering out irrelevant stimuli poses a significant challenge in cognitive capacity. Memory for the to-be-remembered stimulus (henceforth the *target*) tends to be impaired due to the presence of the to-be-ignored stimulus (henceforth the *distractor*) either by the demand for concurrent processing [3, 4] or by the interference from prior experience [5, 6]. Despite the prevalence of multiple streams of incoming information and the importance of successful source memory in daily life, neural correlates of source memory under distraction are relatively poorly understood.

It is well established that successful memory for associations is mediated by activity in the medial temporal lobe (MTL), especially the hippocampus and the parahippocampal cortex for binding of an item and context during encoding [7]. Cortical effects of subsequent source memory have often been reported in the left prefrontal cortex including the ventrolateral prefrontal cortex (VLPFC) and the dorsolateral prefrontal cortex (DLPFC), and the temporo-occipital cortex including the fusiform cortex for visually presented stimuli [8–13]. Based on these findings, it has been suggested that successful encoding of source memory is supported by both associative binding in the hippocampus and semantic elaboration/control in the left prefrontal region, particularly in the VLPFC [13]. However, it has not been well established whether the formation of source memory with a distractor recruits neural activity similar to the activity recruited for source encoding without a distractor or whether the formation of source memory with a distractor calls for additional, distinct activity.

In previous fMRI studies, two brain regions were noted for neural activity of target processing with interference: the left prefrontal cortical region for supporting cognitive control [6, 14, 15] and the hippocampus for resisting interference [16, 17]. These studies focused on the retrieval mechanism with the proactive interference paradigm to identify neural activity resistant to interference effects and indicated the importance of the left prefrontal cortex and the hippocampus in memory with interference. However, the findings from proactive interference studies may not directly apply to the understanding of neural correlates of episodic encoding under distraction because of the difference between interference from previous experience versus interference from current experience. Further, it is unknown whether successful encoding with the distractor employs neural activity similar to that for successful retrieval with the distractor, or whether the effect of distractors in associative memory is similar to the effect of distractors in item memory. The present study addresses the effect of interference due to the concurrent distractor in the formation of an episode with contextual information.

The Stroop task has been a useful experimental paradigm for demonstrating interference effects in task performance, as the incongruent condition includes both the target and the distractor (see [18] for review)^a^. fMRI studies of the Stroop task revealed the involvement of the attentional network against interference in the dorsolateral part of prefrontal and parietal cortices [19–21]. The findings from Stroop task studies imply the role of cognitive control (e.g., attention) in cognitive processing with distractors. Given that source memory is considered to be supported by effortful recollection process, cognitive demand for source memory under distraction may require cognitive control to a different extent than item memory under distraction. Besides, experimental paradigm of interference such as the Stroop task and the picture-word interference (PWI) task showed that a semantic distractor was more interfering to the processing of the target in naming accuracy and reaction times (RTs) than a non-semantic distractor [22, 23]. However, the extant literature is rather lacking regarding the question whether the formation of source memory under distraction would require the involvement of the top-down attentional network as in the interference task paradigm, or if so, whether semantic and non-semantic distractor would exert its interfering effect to source memory in a similar extent while source memory tends to require cognitive control as an effortful process.

The present study employed an fMRI subsequent memory procedure to investigate encoding activity of source memory under distraction due to concurrently presented irrelevant stimuli. For each study trial, a target item (picture) in a perceptual context (color) was presented in one of three conditions: no-distractor, a nonsense letter-distractor, or a word-distractor. Participants were instructed to form an object image incorporating the target and context and to make an ensuing semantic decision on the image for promoting contextual associations. The main research questions included whether the formation of source memory with a distractor recruits neural activity distinct from source memory formation without the distractor, and whether source encoding with the distractor elicits neural activity to a different extent than source memory without a distractor in order to overcome interference effects as in item memory. We hypothesized the following:

(A) Source memory formation under distraction would elicit greater activity in the left DLPFC reflecting cognitive control for target processing, given that previous studies showed the role of DLPFC in imposing an attention set for task-relevant stimuli in interference conditions [19, 24].
(B) The hippocampus would also show enhanced activity for source memory formation, as this region has been suggested for the role in the resistance to interference [16, 17] as well as memory associations between an item and context [9, 25].
(C) Behaviorally, the difference in source recognition between letter-distractor trials versus word-distractor trials would be minimal, as even the formation of source memory would already be cognitively demanding. Therefore, while the presence of a distractor, whether it is semantic or non-semantic, would exacerbate the cognitive load of source encoding, the type of the distractor would not have much additional effects on behavioral performance.

## Results

### Behavioral results

The proportions of studied and new items for each class of response on the source recognition test are displayed in Table 1, along with study RTs. Recognition accuracy measured by *d’* showed that source recognition from all three conditions were above chance: 1.43 [*SE* = .14] for no-distractor; 1.21 [*SE* = .13] for letter-distractor; and 1.16 [*SE* = .13] for word-distractor trials, *t*_[20]_s >2.3, *ps* < .05. A repeated measures ANOVA showed a main effect of distractor on *d’*, *F*_[2,40]_ = 13.6, *p* < .001. Follow-up *t*-tests revealed greater source recognition with no-distractor trials than letter-distractor or word-distractor trials (*t*_[20]_s >3.7, *p*s < .001). However, the difference of *d’* between letter-distractor versus word-distractor trials did not reach significance. In addition, a repeated measures ANOVA on the study RTs sorted by later source recognition judgments showed that study RTs did not differ depending on source judgment or distractor, and there was no interaction.

**Table 1:** **Mean proportions of recognition judgments (SE) and study reaction times (ms) according to study condition and test response**

	Studied item			New item	
Study condition	Source correct	Source incorrect	Misses	CR	FA
Picture-only	.57 (.03)	.30 (.03)	.11 (.02)	.86 (.02)	.13 (.01)
	1395 (62)	1438 (56)	1418 (76)		
Picture-nonword	.49 (.03)	.32 (.02)	.16 (.02)		
	1442 (49)	1527 (64)	1518 (71)		
Picture-word	.47 (.03)	.35 (.02)	.15 (.01)		
	1528 (61)	1557 (76)	2100 (567)		

Note: There were few items for which no recognition judgment was made.

## fMRI results

### Whole brain analysis

The primary goal of the present fMRI study was to investigate neural correlates of successful source memory formation under distraction with irrelevant stimuli. Prior to looking for the effect of distractor on source memory, we first queried *main effects* of source memory and distractor. To examine the effect of source memory, the contrast of source correct greater than source incorrect from all trials was exclusively masked with the interaction of source memory and distractor in order to eliminate the region where source memory differed by distractor type. Encoding activity of successful source memory was found in the left mid DLPFC and superior medial frontal lobe and the right striate cortex and mid temporal cortex, as displayed in Table 2. We also probed the main effect of distractor during encoding. Given that behavioral results showed no difference in source memory between letter- and word-distractor conditions, we collapsed letter- and word-distractor trials and computed the contrast of distractor versus no-distractor. The outcome of this contrast was exclusively masked with the interaction of source memory and distractor to remove the region where the effect of the distractor differed by source memory. As seen in Table 2, extensive neural activity in the regions spanning bilateral occipito-temporal cortices was related to study processing when the target was presented with a distractor, compared to the target without a distractor. Further, the clusters in the left mid/posterior VLPFC and the superior parietal lobule were also associated with study activity under distraction.

**Table 2:** **Main effects of subsequent source memory and distraction**

Coordinates (x y z)			***Z***	# of voxels	Regions	BA
**Source memory (source correct > source incorrect)**						
−54	27	27	4.15	39	L mid dorsolateral prefrontal cortex	46/9
−6	36	42	3.80	34	L superior medial frontal lobe/dorsal anterior cingulate cortex	32
51	−51	−9	3.81	45	R mid temporal cortex	37
6	−96	0	3.91	53	R striate cortex	17
**Distraction (distractor > no-distractor)**						
−42	15	27	3.87	54	L posterior ventrolateral prefrontal cortex	45
−24	−51	42	4.23	50	L superior parietal lobule	7
−36	−93	−9	7.56	548	L temporo-occipital cortex	18/19/20/37
36	−84	−12	5.12	180	R inferior/middle occipital cortex	18/19//20

**Figure 1:**
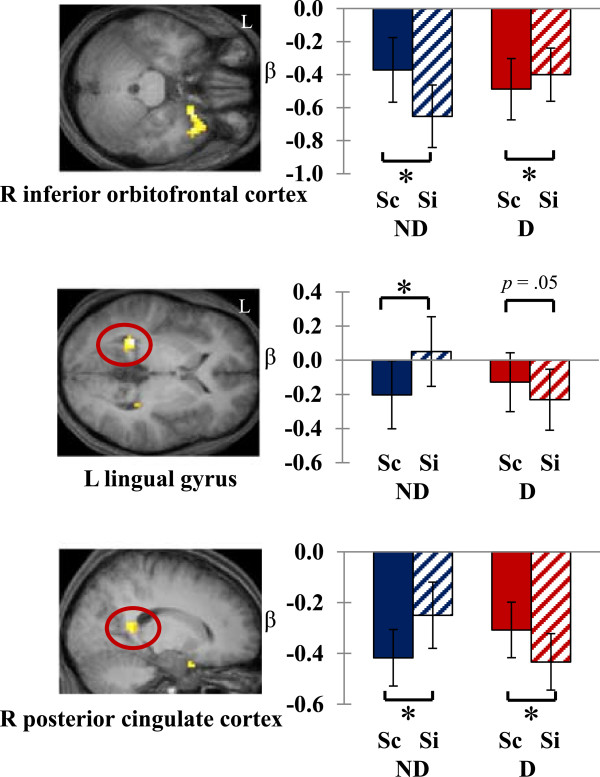
**Brain regions showing the effect of distraction in source memory formation (Sc: source correct, Si: source incorrect, ND: no-distractor, D: distractor).** *p < .05. Error bars depict standard errors of the mean difference across participants.

Next, we queried brain regions showing the *interaction* of source memory formation and the presence of distraction, in order to see the effect of a distractor in source memory. As seen in Figure 1, activity in the inferior-orbital part of the right prefrontal cortex extending to the insula was associated with source memory formation; encoding activity for later source correct items was greater than source incorrect items without distractors, whereas encoding activity for source correct items was smaller under distraction. On the other hand, the left lingual gyrus area and the right posterior cingulate cortex showed decreased activity for the items later remembered with correct source information compared to items without source information in the no-distractor condition. However, when the target items were presented with distractors, the results were in the opposite direction: items that were later remembered but without correct source information tended to show more decreased activity than items with correct source information.

### ROI analysis

**Figure 2:**
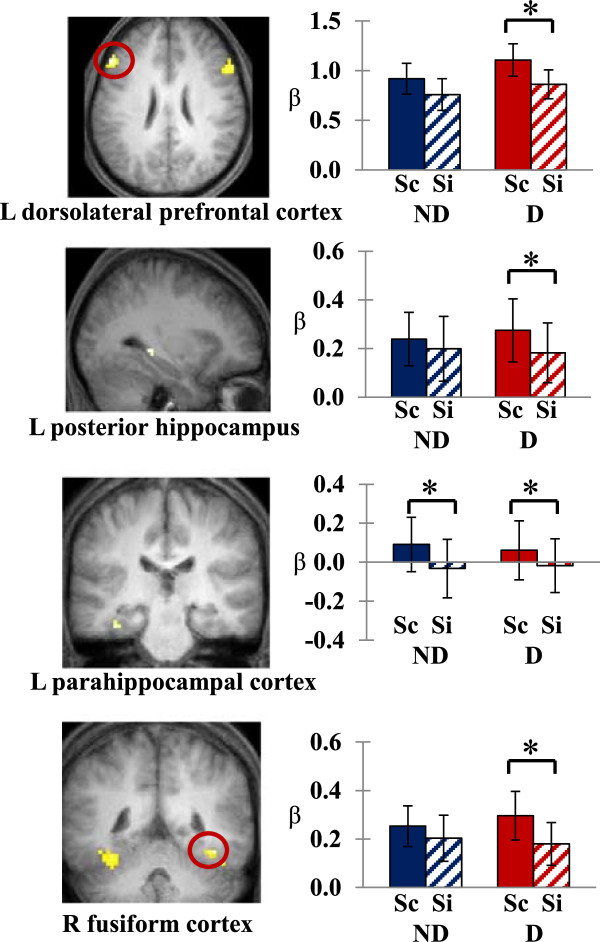
**The effect of distractors in source memory formation in ROI regions (Sc: source correct, Si: source incorrect, ND: no-distractor, D: distractor).** *p < .001. Error bars depict standard errors of the mean difference across participants.

To further investigate source encoding activity under distraction, we performed ROI-based source memory analyses. In light of subsequent memory effects, six bilateral ROIs (prefrontal cortex, MTL, and fusiform) were studied. The ROI analysis revealed clusters that showed effects of the distractor in source memory formation (Figure 2). Notably, the DLPFC showed the difference between hemispheres: a repeated-measures ANOVA by factors of hemisphere (left, right), distractor (no-distractor, distractor), and source accuracy (source correct, source incorrect) on parameter estimates extracted from DLPFC clusters showed interactions of hemisphere x distractor x source accuracy (*F*_[1,20]_ = 7.26, *p* = .01). The left DLPFC showed greater source memory activity under distraction (*t*_[20]_ = 3.69, *p* < .001), while the right DLPFC revealed source encoding effects only in the no-distractor condition (*t*_[20]_ = 3.72, *p* < .001). Additionally, bilateral fusiform cortex regions extended to the inferior temporal gyrus showed enhanced source memory activity in the distractor condition, but not in the no-distractor condition. In contrast, the left anterior VLPFC showed greater activity for encoding of items later remembered with correct contextual information regardless of the presence of a distractor.

In the MTL, activity in the left collateral sulcus extended to the parahippocampal cortex also showed greater encoding activity for source correct items than source incorrect items regardless of whether the target item was presented with or without a distractor. On the other hand, left posterior hippocampal activity was noted for source memory formation only when the target item was presented with a distractor.

## Discussion

The present study was performed to investigate whether a distractor modulates encoding activity for successful source memory. We manipulated the interfering effect of a distractor by presenting no irrelevant stimuli (no-distractor), non-semantic irrelevant stimuli (letter-distractor), and semantic irrelevant stimuli (word-distractor). To the best of our knowledge, the present study is the first to investigate neural correlates of successful source memory formation under distraction from concurrently presented stimuli. By identifying encoding activity of source memory with the distractor and comparing neural activity between source encoding with a distractor versus source encoding without a distractor, we were able to examine neural correlates supporting successful source memory formation under distraction. Subsequent source memory effects under distraction were evident in the left DLPFC, bilateral fusiform cortex/inferior temporal gyrus, and the left posterior hippocampus. These findings provide new insights into the effect of distractors in forming memory associations. Below, we discuss these results and their implications in detail.

### Behavioral findings

As expected, source memory performance was better when the target and context were presented without a distractor than when the target and context were embedded with the distractor; however, source recognition did not differ between the two distractor conditions. It may seem surprising that we did not observe PWI effects from the comparison between letter- and word-distractor conditions, unlike previous studies [26–29]. At a glance, interference task paradigms such as the Stroop task and the PWI task ostensibly predict more distraction in the word-distractor condition than the letter-distractor condition due to the semantic property of the object depicted by a word distractor to the object represented by a target. It should be noted that previous studies measured the PWI effect either by naming latency or the accuracy of the item naming task rather than the accuracy of source recognition. Yet, source memory tasks presumably demand more cognitive resources even without a distractor than an item memory task. In this regard, the current null finding of interference differences between letter- versus word-distractors indicate that the mere presence of a concurrent distractor, regardless of whether it is semantic or non-semantic, may create cognitive load sufficient enough to weaken contextual binding. In other words, the overall effects of the distractor in binding an item with context may have greatly outweighed the difference between semantic versus non-semantic characteristics of distractors with regard to source memory formation.

### fMRI findings

For the main effect of distraction, greater activity for studying stimuli under distraction was identified in the mid and posterior parts of the left VLPFC (pars triangularis). The present findings in the left mid/posterior VLPFC matches with the role of the VLPFC in general cognitive control [14] as study processing under distraction was likely to demand cognitive control compared to study processing without the distractor. Study processing with distractors was also found in the superior parietal lobule, where activity has been noted for perceptual processing of visuospatial stimuli [29], the manipulation of information for online processing [30], and top-down and goal-directed attentional control [31, 32]. Similarly, the fronto-parietal attentional network through the VLPFC and the superior parietal lobule has been identified in previous studies for cognitive processing with distractors [24, 33]. Thus, current findings of the left VLPFC and superior parietal activity indicate that attentional control was most likely called upon for processing the target and context under distraction. Additionally, extensive activity in bilateral occipito-temporal cortices for study processing under distraction reflects visual processing in ventral visual pathways when the pictorial target was embedded with other visual distractors, independent of response accuracy. Further, consistent with previous studies, the main effect of source memory was identified in the left dorsolateral and superior frontal cortex, which are areas that have been noted for their roles in memory associations [11, 12, 34]. Activity in the temporo-occipital cortex was also identified in source memory formation, and this region has been known for perceptual processing for successful associative encoding [8, 35]. These findings suggest that perceptual processing of visually presented targets and context was critical for promoting the formation of successful source memory, as the contextual feature associated with the target item was a visual attribute of color. In fact, the localization of the occipital cortex predictive of successful memory has been reported in previous studies of item and source memory [25, 36–39], and the current finding extends the importance of perceptual processing in source memory formation.

With regard to interaction effects of distractors and source memory, the right orbital prefrontal area to the anterior insula showed decreased encoding activity for items that were later remembered without correct source information in the no-distractor condition while source correct items showed less activity than source incorrect items under distraction. This pattern of findings suggests the possibility that this region is involved in encoding of correct source information in different directions depending on the presence of the distractor. On the other hand, the left lingual gyrus and the right posterior cingulate regions showed decreased encoding activity for items later remembered with correct source information under no distraction; however, the pattern was the opposite under distraction. The lingual gyrus has been noted for the encoding of picture items as memory-related visual processing [40–42]. Considering that visual representational processing of pictorial items with perceptual features was most likely to be critical for successful source encoding, the interaction between source accuracy and distractor in this region indicates that visual processing was sensitive to external distractors, particularly perceptual distractors. The posterior cingulate cortex has been considered as a key node in the default mode network and is involved in learning and memory, such that posterior cingulate activity was modulated by detecting changes, thereby regulating the focus of attention and promoting behavior shifts [43, 44]. The current finding of greater encoding activity for source correct trials in the distractor condition supports the account of distractor detection and consequent regulation in cognitive processing for successful source encoding under distraction, while decreased activity for source correct trials in the no-distractor condition is in line with the conventional pattern of encoding activity in the default mode network area.

Further, the ROI analysis revealed distinctive functions of ROIs in source memory formation under distraction. In the frontal cortex, the DLPFC demonstrated a difference between hemispheres such that left DLPFC was sensitive to source encoding when targets were presented with distractors, while right DLPFC activity contributed to source memory formation regardless of a distractor. The involvement of the left prefrontal cortex in memory formation has been well established in previous studies of memory for single items [8–10, 45, 46] and contextual associations [11, 13, 47, 48]. Successful source memory formation under distraction seems to have required active control to focus attention on the target while filtering out irrelevant stimuli. In this regard, left DLPFC activity most likely reflected control processing for keeping an attention set focused on the target. This type of processing was critical for successful contextual binding under distraction, considering that the need to hold the attention set in order to form memory associations would presumably be critical on a source memory task with distractors. A recent rTMS (repetitive transcranial magnetic stimulation) finding also indicated the importance of left DLPFC activity for self-initiated elaborative encoding by demonstrating that the disruption of left DLPFC activity disrupted memory performance only in the self-initiated encoding condition but not in the instruction-provided condition [49].

On the other hand, the left anterior VLPFC (pars orbitalis) predicted subsequent source memory effects in study trials in both no-distractor and distractor conditions. The left anterior VLPFC has been shown to have a general role in promoting long-term memory formation [11, 12]. In particular, the two process model of the VLPFC [14] distinguishes the role of the left anterior VLPFC in semantic retrieval from the general control in the left mid VLPFC. Our findings are in line with such functional dissociation of the VLPFC, in that successful encoding of pictorial items with the background color could have required semantic processing (e.g., naming the object). Overall, the present findings in the prefrontal cortex indeed demonstrate distinctive functional processing during encoding of contextual associations under distraction with the dorsal-ventral axis in the prefrontal cortex and rostral-caudal axis in the VLPFC.

In bilateral fusiform areas, greater activity was found for source correct trials relative to source incorrect trials under distraction. Since both the target and context were visually presented, perceptual processing of visually presented stimuli likely facilitated the formation of source memory in these conditions. However, a strong visual representation through object processing may have been emphasized in the viability of source memory when the distractors interfered with object processing of the target. In other words, when the distractor was concurrently present, the importance of object processing for forming visual representations may have been even more crucial compared to the condition when no distractor was present.

In the MTL, successful source memory with distractors was predicted in the posterior part of the left hippocampus. The hippocampus has been identified for overcoming interference from overlapping memory representations based on computational models [50, 51] and empirical findings during retrieval [16, 52]. Posterior hippocampal activity with successful source encoding under distraction indicates that the involvement of the hippocampus in memory associations extends beyond the role of distinguishing the target from the lure through pattern separation. The hippocampus contributes to overcoming interference from concurrently presented irrelevant stimuli to form strong item-context associations. This finding adds more weight to the established significance of the hippocampus in episodic memory, such that the hippocampus may play a role in forming source memory by overcoming distraction. In contrast, source encoding activity from the left parahippocampal cortex extended to the collateral sulcus was significant in both no-distractor and distractor conditions. The finding seems to support a general processing of item-feature associations for source encoding in the parahippocampal cortex [13, 53]. Taken together, these findings suggest that successful source encoding under distraction recruits collective neural activity related to attentional control, resistance to interference, as well as contextual binding.

## Methods

### Participants

Twenty-one volunteers participated in the experiment (14 female; 18–26 years old). All of them were right-handed, native English speakers with no reported history of neurological or psychiatric illness. An additional three participants were excluded due to incomplete data. Volunteers were remunerated for participation. Prior to participation, informed consent was obtained in accordance with the requirements of the University of Texas at Arlington and the University of Texas Southwestern Medical Center Institutional Review Boards.

### Experimental materials

The study list consisted of 252 line-drawing pictures denoting objects presented in one of four color squares (blue, green, red, or yellow). Study stimuli were presented in one of three distractor conditions: (A) picture in a color square without distractor (no-distractor); (B) picture in a color square with a nonsense letter-string (letter-distractor); or (C) picture in a color square with a word (word-distractor). Additional file 1: Figure S1 illustrates a schematic of study conditions. A word-distractor used with a picture was not the name of the picture in the study, and the word and picture were not drawn from the same semantic category. The distractor was superimposed on the target picture in the color square. The test list contained a pseudo-random sequence of 252 studied and 126 new pictures, totaling 378 pictures. During test, only pictures were presented without a color square or a distractor. For study and test lists, the same type of the stimulus (e.g., color or distractor) occurred no more than three consecutive times. Study and test lists were separately constructed for each participant. Each stimulus was centrally presented and displayed at a maximum visual angle of 7° × 7° including the square frame. An additional 36 pictures were used for practice.

### Procedure

Participants were given instructions and practice for the task before the experiment proper. In the scanner, each study trial began with a red fixation cross appearing on the screen for 500 ms for warning the upcoming study stimulus. A black/white line-drawing picture in a color square was then displayed for 2000 ms, either without a distractor (no-distractor) or with a distractor (letter-distractor or word-distractor). The study stimulus was replaced by a response prompt for a further 1000 ms, resulting in a stimulus onset asynchrony (SOA) of 3500 ms. Participants were instructed to imagine the object depicted in the picture in the background color and make a pleasantness judgment for the colored object; this encouraged participants to engage in item-context associations. Participants indicated the study judgment by pressing a corresponding button with a finger. The assignment of fingers to responses was counterbalanced across participants. A pseudorandom inter-stimulus interval (ISI) of 2-8 s occurred between trials. The study list was presented across three scan runs that were separated by approximately 2-m breaks. The source recognition test was administered immediately following the study phase in the scanner. A list of test pictures was presented one by one without background color or distractor. Participants were instructed to judge whether each test picture was studied and to indicate in which color the studied picture was presented. Participants responded using a one-step source recognition response with 5 possible options (new, studied-blue, studied-green, studied-red or studied-yellow). Additionally, participants were explicitly instructed to respond with ‘new’ if they were unsure of the study color or the study status of the test item.

### fMRI scanning

A Philips 3 T MR scanner equipped with a 32-channel head coil was used to acquire both T_1_–weighted high-resolution anatomical images (MP-RAGE pulse sequence, 240 × 240 matrix, 1 mm^3^ voxels, sagittal acquisition) and T_2_^*^–weighted echo-planar images (EPIs) (flip angle 70°, 80 × 80 matrix, FOV 24 cm, TR 2000 ms, TE 30 ms, SENSE factor 1.5) per volume. Each volume comprised 33 slices oriented parallel to the AC-PC line (3 mm-thick slice, 1 mm inter-slice gap, 3 mm^3^ voxels) acquired in a descending sequence. Encoding data were acquired in three scanning sessions during the study phase, comprising 450 volumes. An additional five volumes were collected at the beginning of each scan session but discarded to allow for T_1_ stabilization.

### fMRI data analysis

Statistical Parametric Mapping (SPM8, Wellcome Department of Cognitive Neurology, London, UK) run under Matlab R2011b (Mathworks) was used for data preprocessing and statistical analyses. For each participant, functional images were subjected to spatial realignment to the mean image, slice time correction to the middle slice, reorientation, normalization to a standard EPI template based on the Montreal Neurological Institute (MNI) reference brain, and smoothing with an isotropic 8 mm full-width half-maximum Gaussian kernel. The time series in each voxel were high-pass filtered to 1/128Hz to remove low-frequency noise and scaled to a grand mean of 100 across voxels and volumes. T_1_-weighted anatomical images were normalized to the T_1_ template of the MNI brain, and the mean structural image was created across participants. Functional analysis was performed using a General Linear Model (GLM). For each participant, neural activity was modeled with a 3-s duration boxcar function from each study stimulus onset.

For analyses of subsequent memory effects, six events of interest were defined^b^: ‘no-distractor/source correct’ (studied items that were accompanied with correct judgment of study color in the no-distractor condition); ‘no-distractor/source incorrect’ (studied items endorsed as studied albeit with incorrect source judgment or misses in the no-distractor condition); letter-distractor/source correct’ (studied items that were accompanied with correct judgment of study color in the letter-distractor condition); ‘letter-distractor/source incorrect’ (studied items endorsed as studied but with incorrect source judgment or misses in the letter-distractor condition); ‘word-distractor/source correct’ (studied items that were accompanied with correct judgment of study color in the word-distractor condition); and ‘word-distractor/source incorrect’ (studied items endorsed as studied but with incorrect source judgment or misses in the word-distractor condition). Trials associated with omitted responses or wrong key-presses were modeled as events of no interest. The design matrix also included regressors modeling movements, separate scan sessions and the across-scan mean. Parameter estimates for events of interest were computed for each participant using the GLM. Nonsphericity of the error covariance was accommodated by an autoregressive (AR) model, in which the temporal autocorrelation was estimated by pooling over suprathreshold voxels [54]. The parameters for each covariate and the hyperparameters governing the error covariance were estimated using Restricted Maximum Likelihood. Participant-specific parameter estimates for each event of interest were derived and entered into the second level of analysis.

For statistical analyses, we used two complementary analysis approaches. First, we used a standard whole-brain GLM approach. For this whole-brain analysis, we performed a within-subject 3 × 2 ANOVA with factors of Distractor (no-distractor, letter-distractor, word-distractor) and Source memory (source correct, source incorrect) implemented in SPM8. Planned contrasts including pair-wise contrasts (*t*-maps) and interaction contrasts (*F*-maps) were derived from the ANOVA. In order to control Type I-error, the cluster threshold was estimated by using the Monte Carlo simulations implemented in Analysis of Functional Neuroimages (http://afni.nimh.nih.gov/afni) to estimate the minimum cluster size necessary for a cluster-wise corrected significance level of *p* < .05 at a height threshold of *p* < .001. For the whole-brain cortical effect, the cluster-wise corrected significance level of *p* < .05 was thresholded with 31 voxels. The threshold of the exclusive mask identifying voxels where effects were not shared between two contrasts was set at *p* <0.05 for one-sided *t* contrasts and at *p* <0.1 for 2-sided *F* contrasts (note that the more liberal the contrast, the more conservative the exclusive masking procedure). Second, in order to assess the pattern of activation in *a-priori* regions and to complement the whole-brain analysis, we adopted anatomical regions of interest (ROI) approach [55]. As we were specifically interested in the effect of distraction in the formation of source memory, we performed additional ROI analyses on the brain regions where subsequent memory effects were identified from the whole-brain analysis: the left prefrontal cortex, the MTL, and the fusiform cortex [56]. Cortical ROIs were bilaterally derived using the anatomical labels of the Anatomical Automatic Labeling (AAL) atlas [57] implemented in the WFU Pickatalas Tools [58]. For MTL, a manually-drawn mask limited to the hippocampus and the adjacent MTL cortex [59] was used to define the ROI. These ROIs were used as masks with inclusive masking procedures to identify MTL activity. An in the case of the whole-brain threshold, the MTL cluster threshold was estimated by Monte Carlo simulations on AFNI for a corrected significance level of *p* < .05 at a height threshold of *p* < .001. Critical cluster size for the MTL cluster was estimated to 7 voxels. Parameter estimates (β) were extracted from the peak voxels of a cluster for each participant and subjected to group level analyses. The peak voxels of clusters exhibiting reliable effects are reported in MNI coordinates.

## Conclusions

The understanding of episodic memory requires the specification of neural mechanisms involved in forming memory representations when an irrelevant distractor interferes with encoding of the target. Investigating this process is important, as we are surrounded by the demand of encoding associations under distraction. The present study implicates the importance of the posterior hippocampus in forming memory associations under distraction, arguably the most ubiquitous form of learning and memory in modern society. Further, activity in the left DLPFC and bilateral fusiform/inferior temporal regions was recruited for the encoding of successful source memory under distraction, implying a role of general cognitive control and highlighting the value of visual object representations for the viability of source memory under distraction. Finally, the left anterior VLPFC and the left parahippocampal cortex were involved in source memory formation regardless of the presence of the distractor, indicating their general role in source encoding rather than in cognitive control. These sets of neural activity for successful source memory are most likely to reflect the ensemble dedicated to the selection of targets while restraining distraction from irrelevant stimuli during the processing of target-context associations.

## Endnotes

^a^The divided attention paradigm has been used to examine the effect of attentional control in episodic encoding using PET, EEG, or fMRI [60–62]. Although those studies are relevant to cognitive control, the divided attention paradigm requires processing of two targets while the current study paradigm adopts the interference paradigm with a target and a distractor.

^b^Eleven out of 21 subjects had fewer than 12 trials for item miss conditions. Instead of modeling source misses and items misses separately, the two events were combined to a single event, source incorrect, given that both events did not accompany correct source information, following previous studies [12, 63, 64]. A caveat is that this kind of contrast may not reflect memory activity identified from the contrast between source hits versus source misses to the same extent.

## Electronic supplementary material

Additional file 1: Figure S1: A schematic of the study conditions. (PPTX 111 KB)

## Abbreviations

fMRI: Functional magnetic resonance imaging
MTL: Medial temporal lobe
VLPFC: Ventrolateral prefrontal cortex
DLPFC: Dorsolateral prefrontal cortex
PWI: Picture-word interference
RTs: Reaction times
ROI: Regions-of-interest.
