# Supplementary material for: The formation of source memory under distraction
Source: Behav Brain Funct. 2014 Oct 24;10:40. doi: 10.1186/1744-9081-10-40 (PMC4218999; doi:10.1186/1744-9081-10-40)
Supplement: Supplementary file 1 — Additional file 1: Figure S1: A schematic of the study conditions. (PPTX 111 KB) [file 12993_2014_506_MOESM1_ESM.pptx]

## Slide 1
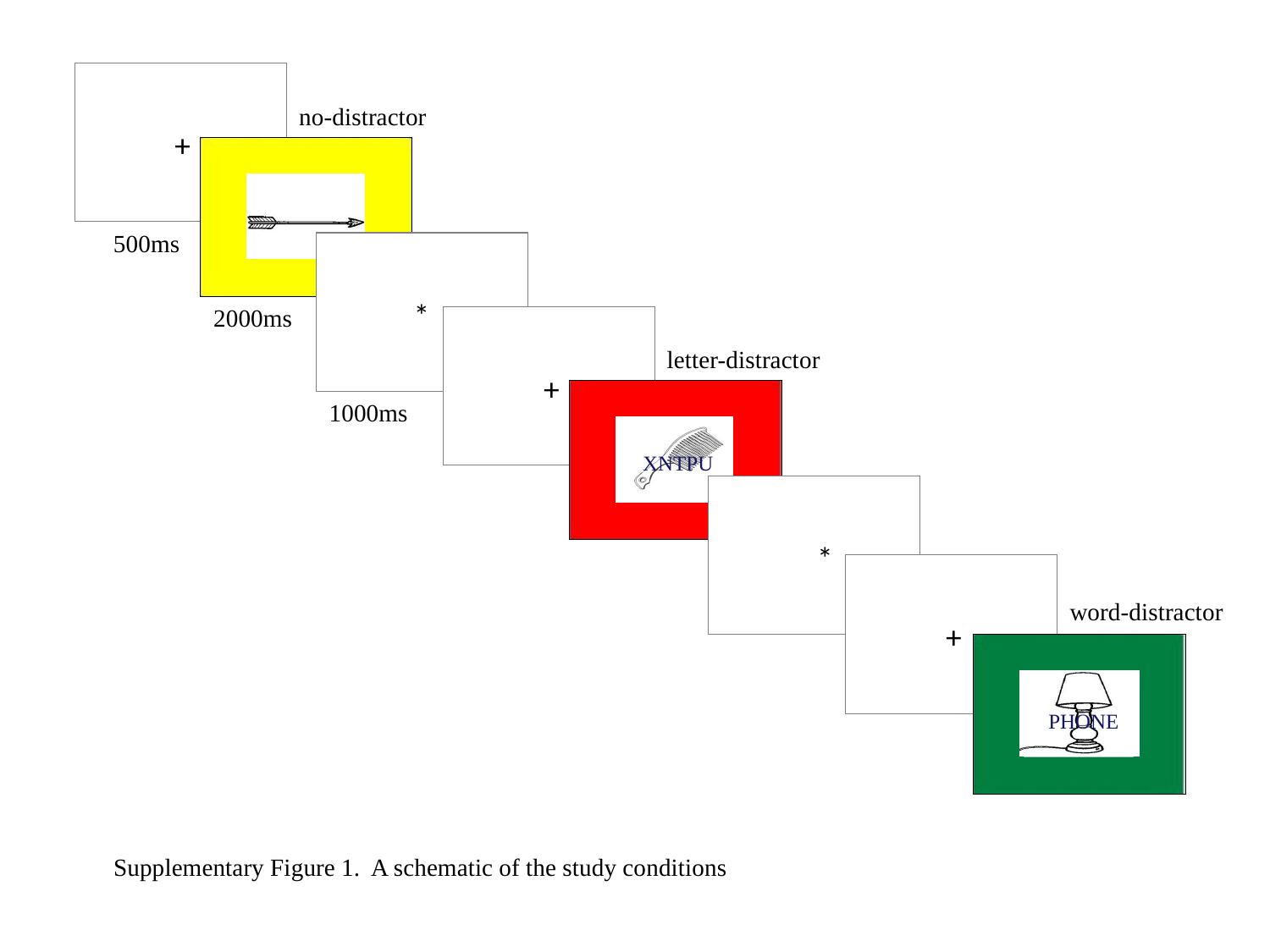

+
no-distractor
*
+
letter-distractor
XNTPU
*
+
word-distractor
PHONE
Supplementary Figure 1. A schematic of the study conditions
500ms
2000ms
1000ms
